# **Assessment of image quality in abdominal CT: potential dose reduction with model-based iterative reconstruction**

**DOI:** 10.1007/s00330-017-5113-4

**Published:** 2018-01-24

**Authors:** Bharti Kataria, Jonas Nilsson Althén, Örjan Smedby, Anders Persson, Hannibal Sökjer, Michael Sandborg

**Affiliations:** 1 Department of Radiology, Department of Medical and Health Sciences, Center for Medical Image Science and Visualization (CMIV), Linköping University, S-581 85 Linköping, Sweden; 2 Department of Medical Physics, Department of Medical and Health Sciences, Linköping University, Linköping, Sweden; 3 School of Technology and Health (STH), KTH Royal Institute, Stockholm, Sweden; 4 Department of Medical and Health Sciences, Linköping University, S-581 83 Linköping, Sweden

**Keywords:** Dose, Computed tomography, Iterative reconstruction, Abdomen, FBP

## Abstract

**Purpose:**

To estimate potential dose reduction in abdominal CT by visually comparing images reconstructed with filtered back projection (FBP) and strengths of 3 and 5 of a specific MBIR.

**Material and methods:**

A dual-source scanner was used to obtain three data sets each for 50 recruited patients with 30, 70 and 100% tube loads (mean CTDI_vol_ 1.9, 3.4 and 6.2 mGy). Six image criteria were assessed independently by five radiologists. Potential dose reduction was estimated with Visual Grading Regression (VGR).

**Results:**

Comparing 30 and 70% tube load, improved image quality was observed as a significant strong effect of log tube load and reconstruction method with potential dose reduction relative to FBP of 22–47% for MBIR strength 3 (p < 0.001). For MBIR strength 5 no dose reduction was possible for image criteria 1 (liver parenchyma), but dose reduction between 34 and 74% was achieved for other criteria. Interobserver reliability showed agreement of 71–76% (*κ*_w_ 0.201–0.286) and intra-observer reliability of 82–96% (*κ*_w_ 0.525–0.783).

**Conclusion:**

MBIR showed improved image quality compared to FBP with positive correlation between MBIR strength and increasing potential dose reduction for all but one image criterion.

**Key Points:**

• *MBIR’s main advantage is its de-noising properties, which facilitates dose reduction.*

• *MBIR allows for potential dose reduction in relation to FBP.*

• *Visual Grading Regression (VGR) produces direct numerical estimates of potential dose reduction.*

• *MBIR strengths 3 and 5 dose reductions were 22–34 and 34–74%.*

• *MBIR strength 5 demonstrates inferior performance for liver parenchyma.*

## Introduction

Technical developments and new applications have led to an increase in the use of computed tomography (CT) in medical imaging and the associated population doses that arise from it [1, 2]. CT contributes up to 70% of the collective effective dose, although it accounts for only 10–15% of the total medical imaging procedures that use ionising radiation [3–6]. Multiphase examinations are more common in abdominal CT, and approximately 30% of CT examinations are abdominal and pelvic, which deliver an effective dose of approximately 6–8 mSv [7]. In recognition of benefits of CT [8], the optimisation of the clinical protocols is motivated to keep the dose as low as reasonably achievable (ALARA principle) [9, 10].

Modern CT equipment presents a number of dose-reduction strategies such as automatic tube current modulation, iterative reconstruction algorithms (IR), dynamic collimation and dose efficient detectors, among others [1, 10, 11].

IR selectively reduces statistical noise in the images thus improving image quality of subtle details, and may facilitate dose reduction. There has been successful improvement in performance of the IR algorithms as they have evolved in the past decade from statistical to model-based algorithms (MBIR) [11, 12]. MBIR, which may be applied at different strengths, perform noise reduction in both raw data and image domains and incorporate physical models to accurately correct for a variety of image degrading effects [13, 14]. Several studies indicate that an increase in the strength of IR allows for larger dose reductions [13, 15, 16]. Evaluations of radiological imaging methods can be performed either by studying their ability to provide correct diagnoses [17] or by visual assessment of well-defined image quality features (visual grading) [18]. However, to our knowledge, there are no studies that directly estimate the dose-reduction potential of the Advanced Modeled Iterative Reconstruction (ADMIRE, Siemens, Erlangen, Germany) algorithm in the clinical setting using pair-wise comparison of images.

The aim of this study was to assess visual image quality between filtered back projection (FBP) and ADMIRE strengths 3 and 5 (out of 5) in abdominal CT, and to estimate the dose-reduction potential of the reconstruction algorithm.

## Material and methods

This was a regional ethical board-approved prospective study conducted at the Centre for Medical Image Science and Visualization (CMIV), Linköping University, Sweden.

A Somatom Force 192-slice dual source CT (Siemens) was used to obtain three data sets of images per patient at 30, 70 and 100% dose levels from a single abdominal acquisition. These were achieved without additional patient exposure as the 30 and 70% tube loads were obtained simultaneously using the dual sources. The acquisition parameters from a standard clinical abdominal protocol are presented in Table 1. Due to a 35.5-cm diameter restriction of the small detector scan field of view (SFoV), ethical approval was obtained for 90 patients as anatomical fit to the smaller SFoV could only be determined after the scan was performed.

**Table 1:** Acquisition parameters for dual source Somatom Force (Siemens, Erlangen, Germany) for tube A and tube B (smaller detector) and Dual Energy Composition (DE comp) set at 0.5 i.e. equal kV weighting for each x-ray tube

Source	U (kV)	Qref (mAs)	Acquisition	Rotation (s)	Pitch	Care Dose 4D	Kernel	Dose level	Slice thickness/Increment (mm)
Tube A+B	120	140	192 × 0.6	0.5	0.6	Yes	Br36	100%	3/2
Tube A	120	98	192 × 0.6	0.5	0.6	Yes	Br36	70%	3/2
Tube B	120	42	192 × 0.6	0.5	0.6	Yes	Br36	30%	3/2

*Qref* quality reference

Patients were informed as to the intent of the study and written consent and approval were obtained. Inclusion criteria were patients over the age of 18 years undergoing a clinical abdominal CT with appropriate patient body habitus determined by visual estimation and use of a calliper to estimate patient size before the scan. Forty patients were excluded due to size and anatomical variations.

Of the 50 examinations, 25 were contrast-enhanced and 25 non-enhanced examinations.

Critical care was taken in patient positioning at isocentre in the gantry. Demographical data such as age, height and weight were also recorded.

### Procedure

The images were anonymised so as to avoid identification of individual patients.

Images at each dose level (30, 70 and 100%) were reconstructed with FBP and ADMIRE strengths 3 and 5. Pairwise visual grading was carried out independently by five radiologists with varying experience (6–20 years), using four modified criteria (C1–C4) from the European guidelines for image quality in abdominal CT [19] together with image noise (C5) and overall image quality (C6) [20] to suit the purpose of this study. The criteria used were as follows:

- C1. Visually sharp reproduction of the liver parenchyma
- C2. Visually sharp reproduction of the pancreas contour
- C3. Visually sharp reproduction of the contours of the kidneys and proximal ureters
- C4. Visually sharp reproduction of the contours of lymph nodes smaller than 15 mm
- C5. Image noise not affecting interpretation
- C6. Overall image quality for diagnostic purposes.

All of the radiologists had 3–4 years’ of experience with SAFIRE strength 3 and one radiologist has been working with ADMIRE strength 3 for a year.

Prior to the study the participating radiologists were coached in grading the different aspects of subjective image quality so as to form a similar understanding of interpretation of the image criteria in order to minimise inter-observer variation. The data sets used in the coaching session were not included in the study population. Each reader rated the criteria in a randomised, blinded and pair-wise approach on DICOM-calibrated (EIZO RX 240) PACS version 17.3 (Sectra, Linköping, Sweden) workstations. The image pairs were graded on a 5-point Likert-type scale (Table 2).

**Table 2:** Ordinal grading scores used for each image criterion in the visual image quality assessment

Grading scores
−2 image on left monitor is better than image on right monitor
−1 image on left monitor is probably better than image on right monitor
0 images on left and right monitors are equivalent
+1 image on right monitor is probably better than image on left monitor
+2 image on right monitor is better than image on left monitor

**Fig. 1:**
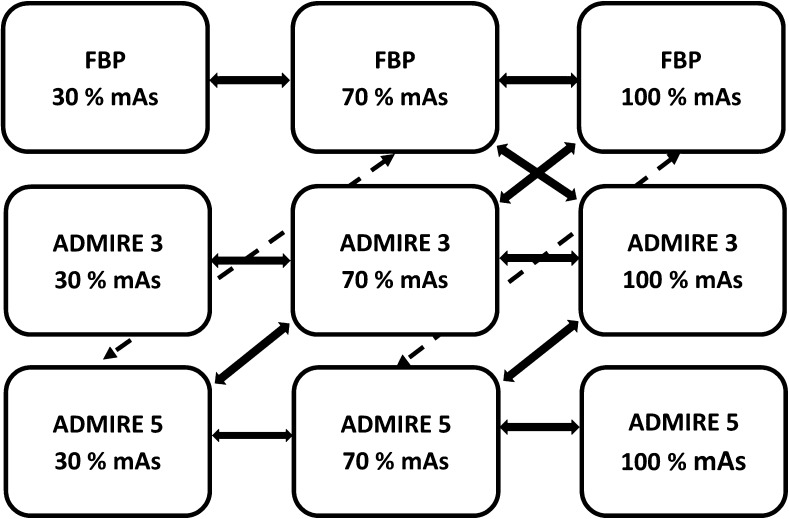
Schematic diagram of the axial image stacks acquired at tube loads, Qref mAs 42 (30%), 98 (70%) and 140 (100%), reconstructed using filtered back projection (FBP) and ADMIRE strengths 3 and 5, with arrows showing the pairwise comparisons performed

Comparison of 12 pairs of image stacks (Fig. 1) per patient resulted in 600 (12 × 50) image pairs per radiologist and a total of 3,000 (600 × 5) image pair assessments. Five of the image pair assessments were replicated to calculate the intra-observer reliability.

### Statistical analysis

Image quality scores were statistically analysed using visual grading regression (VGR) [18]. VGR is an ordinal logistic regression method applied to scores from observer ratings, controlling for dependencies between observers, patients, tube loads and reconstruction methods. Statistical analyses were performed with the software Stata 13.1 (Stata Corporation LP, College Station, TX, USA) using the multi-level mixed-effects ordered logistic regression (*meologit)* command. The regression coefficients describe how the image quality depends on the choice of tube load and reconstruction algorithm, respectively. By relating two of these coefficients to each other, it is possible to estimate the potential dose reduction (*DR*) when replacing one algorithm with another from the equation *DR* = 1 − *e*^−(*b*/*a*)^, where *a* is the regression coefficient for log mAs and *b* that for the iterative reconstruction algorithm [21].

Inter-observer and intra-observer reliabilities were described with the weighted kappa (*κ*_w_) [22] using the *kappa2* command in Stata. The null hypothesis is that neither tube load nor reconstruction method influence perceived image quality. The significance limit was set at p=0.05.

## Results

Of the 50 patients examined, 22 were women, age range 22–90 years (standard deviation (SD) 16.9, mean 64.6) with a body mass index (BMI) of 16.4–27.3 kg/m^2^ (SD 3.0, mean 21.8) and 28 men, age range 44–85 years (SD 10.9, mean 64.8) with a BMI of 16.6–26.2 kg/m^2^ (SD 2.1, mean 23.2). For the study population the CTDI_vol_ ranged from 3.9 to 9.1 mGy (SD 1.3, mean 6.2 mGy), size-specific dose estimate (SSDE) ranged from 6.3 to 12.8 mGy (SD 1.5, mean 8.6 mGy), and dose-length product (DLP) ranged from 161 to 468 mGy.cm (SD 70, mean 292 mGy.cm).

**Fig. 2:**
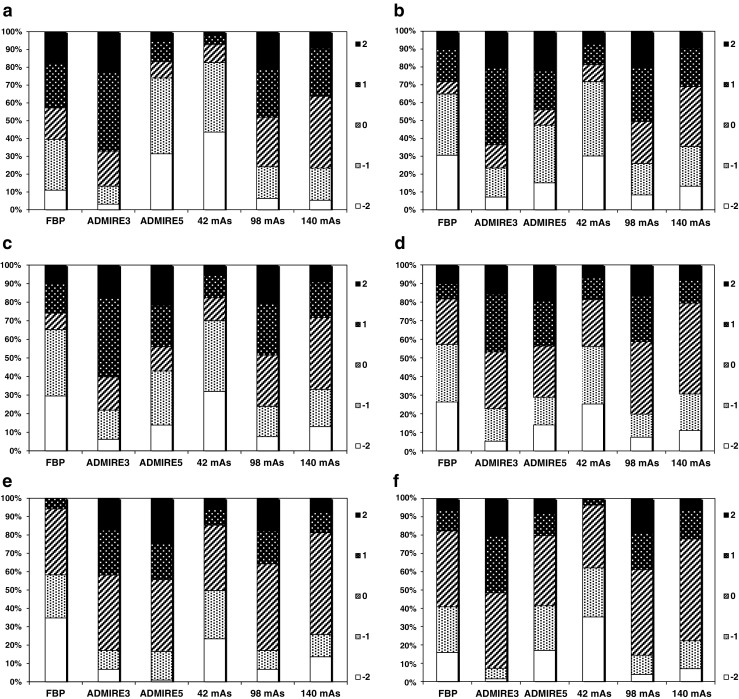
The bars show the percentage of scores assigned to the current image type when compared to other image types. Score 2 indicates that the current image type was rated as superior to the alternative, score 1 that it was rated as probably superior to the alternative, score 0 that the alternatives were rated as equivalent, score –1 that the current image type was rated as probably inferior to the alternative, and score –2 that it was rated as inferior to the alternative. **a** (C1) Favourable versus (vs.) unfavourable scores for image quality criterion 1: Visually sharp reproduction of the liver parenchyma. **b** (C2) Favourable vs. unfavourable scores for image quality criterion 2: Visually sharp reproduction of pancreas contour. **c** (C3) Favourable vs. unfavourable scores for image quality criterion 3: Visually sharp reproduction of contours of the kidneys & proximal ureters. **d** (C4) Favourable vs. unfavourable scores for image quality criterion 4: Visually sharp reproduction of the contours of the lymph nodes < 15mm. **e** (C5) Favourable vs. unfavourable scores for image quality criterion 5: Image noise not affecting interpretation. **f** (C6) Favourable vs. unfavourable scores for image quality criterion 6: Overall image quality for diagnostic purposes

**Fig. 3:**
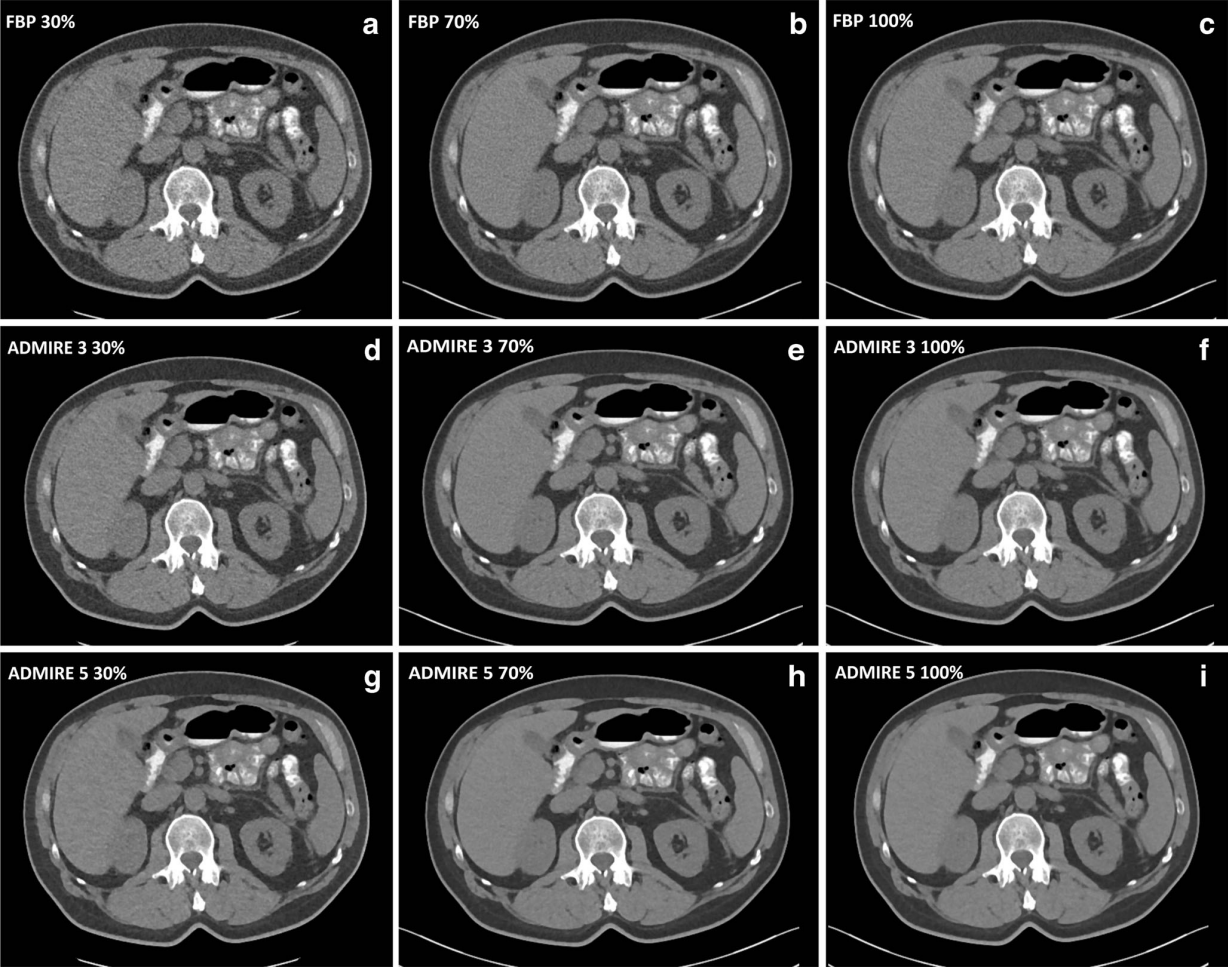
Transverse CT sections at 30, 70 and 100% tube loads reconstructed with (**a**) filtered back projection (FBP) 30%, (**b**) FBP 70%, (**c**) FBP 100%, (**d**) ADMIRE 3 30%, (**e**) ADMIRE 3 70%, (**f**) ADMIRE 3 100%, (**g**) ADMIRE 5 30%, (**h**) ADMIRE 5 70% and (**i**) ADMIRE 5 100% in a 59-year-old male patient with a body mass index (BMI) of 24.8 kg/m^2^

The frequency histograms (Fig. 2) for each criterion show the percentage of favourable versus unfavourable scores (%) with respect to reconstruction algorithm and dose level. Highest scores are seen for quality reference (Qref) mAs 98 (70% dose level) for all image criteria. ADMIRE 3 also tended to yield higher scores when compared with FBP and ADMIRE 5. For ADMIRE 5, scores for overall image quality (criterion 6) were equivalent to FBP and inferior to FBP for criterion 1 (liver parenchyma). Surprisingly the scores for full dose images (Qref mAs 140) are lower than those at the 70% dose level (Qref mAs 98), suggesting that no image quality improvements are obtained with increase in dose. Visual demonstration of image quality in one of the study patients obtained with three tube loads and three reconstruction algorithms are presented in Fig. 3.

The effect on image quality of varying the tube load for each reconstruction algorithm separately is presented in Table 3. A positive value of the regression coefficient in the table indicates that image quality was improved by the change in Qref mAs. A negative value, on the other hand, indicated a reduction in image quality. For FBP, image quality increases with an increase in tube load. For ADMIRE 3 and ADMIRE 5, however, the image quality seems to increase between Qref mAs 42 and 98, but then to decrease for interval Qref mAs 98 and 140. VGR assumes that there is a linear relationship between the log mAs and the logit function score. As this assumption does not hold for the interval between Qref mAs 98 and 140, the subsequent VGR statistical analysis is based on Qref mAs interval 42 to 98.

**Table 3:** Dependency on tube load for different reconstruction algorithms. Visual grading regression (VGR) coefficients for log (mAs) in pairwise comparisons of two quality reference (Qref) mAs values

Criterion	Reconstruction algorithm					
	FBP		ADMIRE 3		ADMIRE 5	
	42 mAs vs. 98 mAs	98 mAs vs. 140 mAs	42 mAs vs. 98 mAs	98 mAs vs. 140 mAs	42 mAs vs. 98 mAs	98 mAs vs. 140 mAs
1. Liver parenchyma	–	3.36**	2.20***	–0.28°	1.49*	–0.37°
2. Pancreatic contours	–	1.89**	2.41***	–0.89*	1.37***	–2.62***
3. Kidneys and proximal ureters	7.25*	2.39*	2.29***	–0.92*	1.46***	–2.20***
4. Lymph nodes < 15 mm in diameter	6.50*	2.09**	1.83***	–1.93***	1.14***	–2.20*
5. Image noise	5.49**	4.70**	3.07**	–	4.79*	1.07°
6. Overall image quality	–	–	3.68*	0.03°	3.74**	–1.27**

* p < 0.05, ** p < 0.01, *** p < 0.001, ° not significant

– Convergence was not achieved with the statistical software available

The VGR result for log mAs and ADMIRE 3 and ADMIRE 5 comparing dose levels at Qref mAs 98 with 42 are presented in Table 4. A significant strong effect of log mAs (p < 0.001) for all six criteria was noted, which indicated an increase in dose for the same algorithm leads to a corresponding increase in image quality. ADMIRE strength 3 resulted in a strongly significant (p < 0.001) increase in image quality relative to FBP for all six criteria. This is reflected in the estimated potential dose reduction ranging from 22% (liver parenchyma) to 47% (image noise). Strong significant results are also seen for ADMIRE 5, criteria 2−6 with a slightly higher dose reduction ranging from 34% (overall image quality) to 74% (image noise), with the exception of criterion 1 (liver parenchyma), for which there was no significant difference between ADMIRE 5 and FBP when assessing the delineation of liver parenchyma.

**Table 4:** Visual grading regression (VGR) coefficients for all six criteria with estimated dose reduction values for comparison of images reconstructed with ADMIRE 3, ADMIRE 5 and filtered back projection (FBP) for dose interval between quality references (Qref) mAs 98 and 42

Criterion	Regression coefficients			Estimated dose reduction (95% confidence limits)	
	log (mAs)	ADMIRE 3	ADMIRE 5	ADMIRE 3	ADMIRE 5
1. Liver parenchyma	2.28***	0.57***	–0.08°	22% (11%; 33%)	−
2. Pancreatic contours	2.00***	0.92***	1.73***	37% (26%; 48%)	58% (53%; 63%)
3. Kidneys and proximal ureters	2.21***	1.11***	2.09***	40% (31%; 49%)	61% (57%; 66%)
4. Lymph nodes < 15 mm in diameter	1.72***	1.05***	1.93***	46% (35%; 57%)	67% (63%; 72%)
5. Image noise	2.38***	1.50***	3.16***	47% (39%; 55%)	74% (71%; 76%)
6. Overall image quality	2.69***	1.06***	1.10***	33% (24%; 41%)	34% (27%; 40%)

***p < 0.001, ° not significant

Comparing results between ADMIRE 3 and ADMIRE 5 in relation to FBP (Table 4), criteria 2–6 suggest that the dose reduction potential is higher with increase in strength. However, for ADMIRE 5 criterion 1 (liver parenchyma) there was no dose reduction possible. Image noise decreases with increase in IR strength, which is reflected by the slightly higher estimated dose reduction for ADMIRE 5 compared to ADMIRE 3.

When comparing ADMIRE strength 3 with ADMIRE strength 5 (Table 5), the regression yielded significant (p<0.001) values for most criteria when dose levels for the same algorithm strength are compared, confirming that ADMIRE 5 is superior to ADMIRE 3. However, for criterion 1 (liver parenchyma) the negative coefficient value (−0.98) indicates that image quality in ADMIRE 5 is significantly lower compared to ADMIRE 3 and hence does not allow for any further dose reduction. Also, overall image quality with a regression coefficient value of −0.85 was not significant. For the criteria assessed, ADMIRE strengths 3 and 5 are superior when compared to FBP individually except for criterion 1 (ADMIRE 5). When it comes to delineation of the liver parenchyma and overall image quality, ADMIRE 5 was inferior to ADMIRE 3 with no dose reduction possible.

**Table 5:** Visual grading regression (VGR) coefficients for all six criteria with estimated dose reduction values for comparison between ADMIRE 3 and ADMIRE 5 in the dose interval quality references (Qref) mAs 98 and 42

Criterion	Regression coefficients		Estimated dose reduction (95% confidence limits)
	log (mAs)	ADMIRE 5 Reconstruction	
1. Liver parenchyma	1.88***	–0.98***	–68% (–102%; –35%)
2. Pancreatic contours	1.84***	0.61***	27% (18%; 37%)
3. Kidneys and proximal ureters	2.06***	0.77***	31% (23%; 39%)
4. Lymph nodes < 15 mm in diameter	1.49***	0.68***	37% (26%; 47%)
5. Image noise	2.42***	1.66***	50% (45%; 55%)
6. Overall image quality	3.18***	–0.85°	−

***p < 0.001, ° not significant

There were marginal differences in the results of the VGR analysis between the groups of patients who received intravenous contrast material and those who did not (data not shown).

The inter-observer agreement was fair, 71–76% with *κ*_w_ ranging from 0.201 (confidence interval (CI) 0.175–0.228) to 0.286 (CI 0.258–0.314). The intra-observer *κ*_w_ values ranged from 0.525 (CI 0.209–0.840) to 0.783 (CI 0.577–1.021), showing a moderate to substantial agreement between 82 and 96% for all the criteria.

## Discussion

In radiology, several analysis methods can be used to describe image quality. The receiver operating characteristic (ROC) analysis method is used to evaluate and compare diagnostic performance [17, 23]. When determining potential dose reduction in the optimisation process, visual grading experiments may be useful [21, 24]. VGR seems to be the only analysis method that produces direct numerical estimates of potential dose reductions for new acquisition, reconstruction and post-processing techniques while image quality is maintained [25].

The present study aimed to estimate the dose reduction potential of ADMIRE strengths 3 and 5 compared with FBP in a standard-dose abdominal CT. It suggests that ADMIRE while preserving image quality allows for a dose reduction relative to FBP of 22−47% (ADMIRE 3) for all criteria assessed, and 34−74% (ADMIRE 5) for criteria 2–6 with the exception of liver parenchyma visualisation. Similar results are reported by Greffier et al, who studied the performance of SAFIRE strengths 1–5 compared to FBP in two data sets at 30 and 70% dose levels. They concluded that a 40–60% reduction in dose is possible [26]. Gordic et al. [27] evaluated both quantitative and qualitative image quality parameters in abdominal CT using ADMIRE. Results from their study showed an improved image quality with lower noise when comparing ADMIRE with FBP, where the amount of noise reduction (53% for ADMIRE 5) could be translated to a reduction in radiation dose (e.g. reduction in effective mAs). However, their study differs from the present study in that they evaluated visibility of only small structures such as small blood vessels, adrenal glands and lymph nodes.

In the present study, the VGR analysis in Table 3 revealed that the 70% dose level (Qref mAs 98) scores were, for the iterative algorithms, higher than full dose (Qref mAs 140) scores for all image criteria. This was an unexpected finding. For FBP, the results were in agreement with the general rule that image quality increases with increasing tube load. For the iterative reconstruction algorithms, one might speculate that a certain amount of noise is required for the algorithm to work optimally. It should be noted that due to technical advancements the Somatom Force scanner has been found to give better image quality than other Siemens equipment [28]. A possible conclusion is that the 70% dose level provides images of sufficiently high image quality. Thus, one can optimise the standard clinical abdominal protocol for the Somatom Force using ADMIRE 3 by reducing the tube load from Qref mAs 140 to 98 without changing the strength of the algorithm. However, as demonstrated in Table 5, by replacing ADMIRE 3 with ADMIRE 5, further dose reduction can be achieved for certain aspects of the image quality, but not all.

With ADMIRE strength 5 there is still a problem as non-linear effects of IR lead to smoothing of the anatomical features and a change in appearance of the anatomy in the images [29]. Mieville et al. [30] reported a change in the appearance of the MBIR images. Certain small objects that were not identified on the FBP images were visualised on the low-dose MBIR images. Suboptimal performance when evaluating small or subtle abdominal structures (i.e. common bile duct, adrenal glands and pancreatic duct) was also reported by Padole et al. [31] when comparing FBP with reduced dose MBIR and Adaptive Statistical Iterative Reconstruction (ASIR). This compromise in the visibility of structures could be attributed to the blotchy, pixelated and plastic-like appearance of the images. The liver parenchyma is a low-contrast object and although image quality is improved using MBIR, it is possible that no improvement is seen in detection of low-contrast details as was the case in the phantom study of Euler et al. [32]. Contrary results were presented by Solomon et al. [13] who studied low-contrast detectability using ADMIRE. There is a possibility that assessing low-contrast detectability *in vivo* is different to phantom images as the task of clinical assessment by a radiologist, who has access to full image data and patient information, is relatively complex compared to the simple task of assessing subtle lesions in a phantom. Solomon et al. [33] used a similar method to the present study to estimate the dose reduction potential of SAFIRE relative to FBP. They also investigated virtual liver lesion detectability in hybrid images. Although their study was simple compared to clinical reality, such experiments do provide valuable information about how different algorithms render the same lesion differently. In the present study, reader confidence in determining the visually sharp reproduction of liver parenchyma was lower for ADMIRE 5 than for FBP. Change in image texture due to the denoising properties of the reconstruction algorithm influences the potential dose reduction depending on the diagnostic task [30]. This may explain the slightly lower estimated dose reduction for ADMIRE 3 and the non-significant result for ADMIRE 5 when assessing the liver parenchyma.

On the other hand, anatomical contour assessments were not a problem even though the higher strength images were smoother in appearance. As our readers pointed out during the coaching session, when intra-abdominal fat is present, the delineation of contours is further enhanced. This was true for the assessment of proximal ureters, lymph nodes and pancreas. However, in patients with low BMI, when a smaller amount of intra-abdominal fat is present, the delineation of contours can be difficult [34].

Low kappa values were seen for inter-observer agreement between all five readers. It is not unusual that there is a variation in perception of image quality among radiologists as viewing strategies differ depending on the approach [35]. The intra-observer agreement in the present study was less than 100%, indicating variation in perception for the same reader at different points in time [36].

Future research is indicated as there is some scepticism among radiologists in using higher strengths of IR. This is mostly related to the change in image texture, due to substantial noise reduction, possibly affecting diagnostic confidence. When comparing delineation of structures in different planes, Mieville et al. [30] reported an improvement in detection of small structures in the coronal plane compared to the axial plane. It would be of interest to study if higher strengths of the algorithm could be made more clinically acceptable with a combination of IR and other post-processing methods. This might increase diagnostic confidence in IR images of higher strength and allow for further dose reductions.

The major limitation of our study was exclusion of overweight patients due to size limitations of the small detector, hence the estimated dose reduction is limited to the patients with a BMI of up to 27.3 kg/m^2^. Image quality might be inferior as noise increases in overweight patients with a BMI > 28, possibly leading to less or no dose reduction [24, 37]. Since there was a variation in patient body habitus, not all scans fitted the 35.5-cm diameter. Some of the patient anatomy, mostly in the pelvic region, slightly exceeded this limit, which may have affected the image quality. However, there were no anatomical image criteria present in this region, except for overall image noise and image quality. It is therefore unlikely that this would have affected the results. There are many image acquisition parameters that affect image quality. We have studied only change in tube load (mAs) and image reconstruction. Hence the study protocol differs from the clinical protocol as a fixed kV was used for both x-ray tubes without automatic adjustment of tube potential (Care kV). Visual grading is an easy and inexpensive method to assess image quality. However, it assumes that whenever normal anatomy is sharply reproduced, the same will apply to pathology. The extent to which this assumption is correct is generally not known. As reconstructed images with MBIR strengths 3 and 5 and FBP all have different appearances, it is difficult to perform a true blinded evaluation of subjective criteria [38]. This enhances the need for further research and analysis of objective image quality parameters to support the subjective findings of this study.

## Conclusion

The model-based iterative reconstruction algorithm ADMIRE showed improved image quality compared to FBP. A positive correlation between ADMIRE strength and increasing potential dose reduction was found for the majority, but not all, of the image criteria.

## Abbreviations

ADMIRE: Advanced Modeled Iterative Reconstruction
ALARA: As low as reasonably achievable
CT: Computed tomography
FBP: Filtered back projection
IR: Iterative reconstruction
kV: Kilovolt
MBIR: Model-based iterative reconstruction
mAs: Milliampere-second
mSv: Millisievert
Qref: Quality reference
VGR: Visual Grading Regression
